# Phenolic compounds and bioactivity evaluation of aqueous and methanol extracts of *Allium mongolicum* Regel

**DOI:** 10.1002/fsn3.926

**Published:** 2019-01-28

**Authors:** Wanyu Wang, Jiao Li, Huizhen Zhang, Xiaokai Wang, Jianming Fan, Xiaofeng Zhang

**Affiliations:** ^1^ Department of Nutrition and Food Hygiene College of Public Health Zhengzhou University Zhengzhou China

**Keywords:** *Allium mongolicum* Regel, angiotensin‐converting enzyme, antioxidant activity, pancreatic lipase, α‐glucosidase

## Abstract

*Allium mongolicum* Regel (AM), widely distributed in western China, is a traditional Mongolian medicine herb. Two different solvents as water and methanol were used to extract AM, and their antioxidant capacity and inhibitory effects against key enzymes related to metabolic syndrome were assessed. The antioxidant capacity was evaluated through the assay of radical scavenging ability on DPPH and ABTS and reducing power assays. In addition, the total phenolic content and total flavonoids content were quantificated and analyzed. Aqueous extract, having higher phenolic content (10.20 mg GAE/g DW) and flavonoid content (4.02 mg QE/g DW), showed better antioxidant and inhibitory effects against lipase and angiotensin‐converting enzyme (ACE); as for α‐glucosidase, the extract made by methanol showed better ability. In general, the aqueous extract of *A. mongolicum* Regel has the potential to be used as a functional food or nutraceutical in prevention and treatment of obesity and hypertension due to the high antioxidant and sound inhibitory potential against vital enzymes relevant to obesity and hypertension.

## 1 INTRODUCTION

*Allium mongolicum* Regel is a perennial plant which belongs to the genus *Allium* of the *Liliaceae* family that grows in high altitude desert, an important Mongolian traditional medicinal herb distributed in Inner Mongolia Autonomous Region and western China (Muqier et al., 2017). According to the Mongolian medicine records, *A. mongolicum* Regel has various special effects such as lowering blood pressure, hypolipidemic, stimulating the appetite, replenishing kidney, and aphrodisiac. As a characteristic vegetable with unique flavor and high nutritional value in desert, *A. mongolicum* Regel is commonly used to please seasoning and assisted to cook delicious dish, providing health benefits to human body. The aqueous extracts of *A. mongolicum* Regel have been shown certain antioxidant effect on mutton sausages (Li, Luo, Fan, & Jv, 2016). Up to now, studies about *A. mongolicum* Regel were mainly focused on animal cultivation and improvement on meat products.

The usage of natural phytochemicals as dietary intervention has received a great deal of attention as a potentially important concept in therapeutic intervention and public health (Hwang et al., 2018). Natural phenolic compounds have been suggested to be one of the interesting secondary metabolites due to their outstanding effects on clinical disease (Lopes et al., 2018). It was indicated that the extracts that contains high amount of polyphenolic obtained from herbs had effects in modulating the activity of selected digestive enzymes, such as lipase, a‐glucosidase, and angiotensin‐converting enzyme (ACE) (Sakulnarmrat & Konczak, 2012). Natural products may play a vital role in blocking the occurrence and development of many chronic diseases such as obesity, diabetes, and hypertension through regulating of various trails, including intestinal catabolism of complex carbohydrates and fat absorption (Perez, Zampini, Alberto, & Isla, 2018).

Oxidative stress, a condition of disturbance in the balance between the formation of reactive oxygen species (ROS) and the antioxidant defense system, performs as a critical part in the progression of various disease, such as type 2 diabetes, obesity, and cardiovascular disease (Bernatoniene & Kopustinskiene, 2018; Kumar Singh & Patra, 2018). Antioxidant, a kind of substance that protect body cells from harmful oxidative, is demonstrated to reduce the damage of oxidative stress on living tissues, hinder the processes of aging, and prevent the progressions of many diseases from happening (Kim et al., 2017; Zhang, Li, Lin, & Li, 2018).

Metabolic syndrome, a cluster of some metabolic disorder, including insulin resistance, dyslipidemia, hyperglycemia and hypertension (Huang, 2009). It is suggested that metabolic syndrome is an early pathological features of chronic disease like type 2 diabetes, which is characterized by increase of glucose levels following food intake (Hung et al., 2017). The inhibitory effects on key enzymes related to carbohydrate hydrolyzes, which located in the small intestine, were thought to be an effective strategy to control the condition of postprandial hyperglycemia (Subramanian, Asmawi, & Sadikun, 2008; Tucci, Boyland, & Halford, 2010). α‐glucosidase, one of the vital carbohydrates enzymes, could catalyze the disaccharides and oligosaccharides degraded to absorbable monosaccharides, which facilitates an uptake of glucose into the blood stream. Therefore, the inhibition effects on the activity of intestinal α‐glucosidase could decreases the glucose digestion and absorption in intestinal from starch and sucrose hydrolysis, thereby reducing the postprandial blood glucose levels. It was demonstrated that the condition of postprandial blood glucose levels could be reduced by inhibiting the activity of α‐glucosidase not only in vitro but also in vivo (Kim et al., 2018).

Obesity, due to an imbalance between energy intake and expenditure, is a major preventable risk factor in the development of metabolic syndrome (Jimenez‐Aspee et al., 2017). The inhibition on digestion and absorption of dietary nutrients is an effective method to the prevention and treatment of obesity. Pancreatic lipase is a key enzyme in the process of dietary fat absorption, which is responsible for 50%–70% of the hydrolysis of dietary triglycerides into monoacylglycerides and free fatty acids, finally been absorbed by enterocytes (Sergent, Vanderstraeten, Winand, Beguin, & Schneider, 2012). Therefore, the inhibitory effect on the activity of lipase is proposed to be useful in the management of blood fat. ACE, another enzyme that have an important impact on metabolic syndrome, plays a pivotal role in the regulation of blood pressure (Villiger, Sala, Suter, & Butterweck, 2015). ACE belongs to the class of zinc metal proteases that catalyzes the conversion of angiotensin I to a potent vasoconstrictor angiotensin II and also promotes the degradation of the vasodilator bradykinin (Paiva, Lima, Neto, & Baptista, 2017). Plenty of synthetic drugs, such as captopril, that used in the treatment of hypertension were ACE inhibitors. Nevertheless, these drugs may cause serious adverse side effects (Lin, Lv, & Li, 2012; Luo et al., 2017). Recently, searching for ACE‐inhibitor from natural plants has been attacked much attention. Several plants in *Liliaceae* family from *Allium* were found as a potential effective remedy of hypertension possibly due to the inhibitory effects on ACE (Oboh, Ademiluyi, Agunloye, Ademosun, & Ogunsakin, 2018).

The potential value and application of *A. mongolicum* Regel lack supporting scientific data, and its use as drugs is based solely on traditions that have perpetuated for generations. For that reason, the aim of the present study was to evaluate the phenolic profile of aqueous and methanol extracts of *A. mongolicum* Regel, and the bioactive properties were explored in terms of antioxidant potential and inhibitory effects against key enzymes relevant to the principal components of metabolic syndrome.

## 2 MATERIALS AND METHODS

### 2.1 Sample

*Allium mongolicum* were collected from Alashan in Inner Mongolia, China, in September 2017. The fresh samples were cleaned and shade dried at 25°C, then dried in vacuum freeze dryer for 24 hr. The dried samples were grinded and defatted by carbon tetrachloride (w/v = 1:10, 24 hr) at 25°C. Defatted flours were sealed and stored in the dark, at 4°C until used.

### 2.2 Preparation of sample extracts

The air‐dried defatted powders of sample (10 g) were soaked in 100 ml pure water and methanol (100%) solvents (1:10, w/v), respectively, at 25°C for 24 hr under dark for twice. The extracts were centrifuged at 5381 *g* for 5 min, and then the supernatants were evaporated under vacuum till the solution volume left was 20 ml. Extract solutions were identified as 500 mg/ml and kept stored in dark at 4°C until further analysis.

### 2.3 Standards and reagents

4‐Nitrophenyl Laurate were purchased from TCL (Shanghai, China). 2,2‐Diphenyl‐1‐picrylhydrazyl (DPPH), 2,2′‐azinobis (3‐ethyl‐benzothiazoline‐6‐sulfonic acid) diammonium salt (ABTS), 2,4,6‐tri (2‐pyridyl)‐s‐triazine (TPTZ), N‐Hippuryl‐His‐Leu hydrate powder (HHL), the Folin–Ciocalteu phenol reagent, lipase from porcine pancreas, ACE (from rabbit lung), and α‐Glucosidase from Saccharomyces cerevisiae were purchased from Sigma Chemical Co (St. Louis, MO, USA). 6‐Hydroxy‐2,5,7,8‐tetramethylchromane‐2‐carboxylic acid (Trolox), 4‐N‐trophenyl‐α‐D‐glucopyranoside (PNPG), Standards of gallic acid and quertein, Triton X‐100, and Tris‐HCL were purchased from Aladdin (Shanghai, China). Other reagents were all of analytical grade.

### 2.4 Quantification of total phenolic content

The total phenolic content (TPC) of each extract was assessed in adherence to the colorimetric Folin–Ciocalteu method reported by Ainsworth and Gillespie (2007). Briefly, 100 μl sample or gallic acid solution which dissolved in methanol at different concentration was mixed with 200 μl of 10% Folin–Ciocalteu reagent, then 800 μl Na_2_CO_3_ (700 mM) was added, and the mixture in assay tubes was incubated at 25°C for 2 hr. Finally, 200 μl mixture was transferred to a clean 96‐well microplate, and the absorbance was quantified by a microplate reader (SpectraMax M2 Molecular Devices USA) at 765 nm. Gallic acid was used as reference standard by using seven‐point analytical curves (measurements in duplicate), with concentrations ranging from 0–500 μg/ml (*R* ^2^ > 0.999). The results were expressed as mg Gallic acid equivalents (GAE)/g dry weight (DW).

### 2.5 Quantification of total flavonoid content

The TFC was ascertained using the classical colorimetric method as described by Arvouet‐Grand, Vennat, Pourrat, and Legret (1994). Briefly, 100 μl of 2% aluminium trichloride (AlCl_3_) in methanol was mixed with the same volume of the sample extracts. Absorption readings at 415 nm were taken after 10 min against a blank sample consisting of a 100 μl extract solution with 100 μl methanol without AlCl_3_. The calibration curve was prepared using various concentrations of quercetin (0–250 μg/ml) dissolved in methanol solution. TFC results were shown as mg quercetin equivalent (QE)/g DW.

### 2.6 DPPH radical scavenging assay

The DPPH assay was performed by the use of 96‐well microplate according to Cheng’ method with some modifications (Cheng, Moore, & Yu, 2006). The mixed solution was measured at 517 nm to evaluate the absorbance using a microplate reader (SpectraMax M2 Molecular Devices USA). The radical scavenging property was assessed by measuring the decrease of its absorbance. Eventually, the results were presented by the form of IC_50,_ that is, the necessary amount of antioxidant to decrease the initial DPPH concentration by 50% and the time taken to reach the steady state to IC_50_ concentration (Antolovich, Prenzler, Patsalides, McDonald, & Robards, 2002). DPPH radical scavenging activity was calculated using the equation below: Scavenging activity (%) = [1−(A_sample_−A_sample blank_)/A_control_] × 100.

The standard curve was established using various concentrations of trolox in methanol. The trolox equivalent antioxidant capacity (TEAC) of relative DPPH scavenging capacity for each sample was showed as mg trolox equivalent (TE)/g DW.

### 2.7 ABTS radical scavenging assay

The ABTS assay was determined as described by Re et al. with slight modifications (Ali, Jung, Jannat, Jung, & Choi, 2016). The stock reagent solution was prepared by mixed 7 mM ABTS solution with 2.45 mM potassium persulfate solution (1:1, v/v). The working solution was made by incubating the stock solution at 25°C in dark for 12–16 hr until the reaction was complete. The resulting ABTS radical solution was diluted with ethanol to an absorbance of 0.70  ±  0.02 at 734 nm for measurement. A volume of 20 ml test samples at different concentrations were added to 180 μl of ABTS^+^˙ working solution. The mixture was incubated for 3 min at 25°C in the dark, and the absorbance of resulting solution was measured at 734 nm using a microplate reader (SpectraMax M2 Molecular Devices USA). Scavenging activity of ABTS radical was calculated using the equation below:

$$\text{Scavenging activity}\;\left(\%\right)=[1-\left(A_{\mathrm{sample}}-A_{\mathrm{sample}\;\mathrm{blank}}\right)/A_{\mathrm{control}}]\times 100$$

The standard curve was established using various concentrations of Trolox in methanol. The TEAC of relative ABTS scavenging capacity was expressed as mg TE/g DW.

### 2.8 Ferric reducing antioxidant power (FRAP) assay

The ferric‐reducing power antioxidant potential was measured on the basis of method described by Benzie and Strain (Benzie & Strain, 1996) with some minor modifications. The mechanism was based on the reduction of the Fe^3+^–TPTZ complex to the ferrous form (Fe^2+^) at low PH; the latter forms a blue complex (Fe^2+^/TPTZ), which increases the absorption at 593 nm. In summary, the FRAP working solution was prepared by mixing acetate buffer (0.3 M pH 3.6), a solution of 10 mM TPTZ in 40 mM HCl, and 20 mM FeCl_3_ at 10:1:1 (v/v/v). Forty microliter of sample extract on different concentration was placed in each well of a 96‐well plate and mixed with 260 μl of the FRAP working solution. The absorbance was measured by a microplate reader at 593 nm after incubated in dark at 37°C for 30 min. Standard curve was prepared using different concentrations of FeSO_4_ solution. All tests were performed in triplicate, and results were reported as μmol of Fe^2+^ equivalents per g of DW.

### 2.9 α‐ glucosidase inhibitory assay

The procedure of α‐glucosidase inhibitory activity was performed in accordance with a previous study (Kang, Song, & Zhang, 2010). Briefly, diluted samples at various concentrations (8 μl) were mixed with 112 μl of phosphate buffer (0.1 M, pH 6.8) and 20 μl of 0.2 U/ml α‐glucosidase solution (in 0.1 M, pH 6.8 PBS), mixed and incubated at 37°C for 15 min, and then, 20 μl of 2.5 mM PNPG solution (in 0.1 M, pH 6.8 PBS) was added. The mixture was processed at 37°C for 15 min and stopped by adding 80 μl of 0.2 M Na_2_CO_3_ solution. The absorbance of resulting solution was measured at 405 nm. The inhibitory capacities were calculated according to the following formula: [1−(A_405sample_−A_405sampleblank_)/(A_405control_−A_405controlblank_)] × 100%.

### 2.10 Lipase inhibitory assay

The inhibitory properties of AM extracts were assessed according to McDougall, Kulkarni, and Stewart (2009) with slight modifications. *p*‐nitrophenyl laurate (*p*NP laurate) was the substrate of this reaction which was hydrolyzed by lipase to *p*‐nitrophenol (*p*NP). Lipase from porcine pancreas Type II was dissolved in ultrapure water at 10 mg/ml; the supernatant was then used as the enzyme source for subsequent experiments after centrifugation at 11430 *g* for 8 min. Solution of 100 mM Tris (pH 8.2) was the buffer of this assay. The substrate stock was 0.08% w/v *p*NP laurate dissolved in 5 mM sodium acetate (pH 5.0) containing 1% Triton X‐100 and was heated in boiling water for 3 min to aid dissolution, mixed well, then cooled to room temperature. In brief, 50 μl sample, 150 μl lipases, and 350 μl assay buffer were mixed and incubated in 37°C for 10 min, and then 450 μl substrate solution was added to start the reaction. After incubated in 37°C for 2h, the reaction mixture was centrifugated at 14,000 rpm for 3 min, and then 200 μl of it was transferred into a 96‐well plate. Finally, read the mixture solution at 405 nm. The pancreatic lipase inhibitory rate was represented as % inhibition according to the formula: [1−(A_405sample_−A_405sample blank_)/(A_405control_−A_405control blank_)] × 100%.

### 2.11 ACE‐inhibitory activity

As for the evaluation of ACE inhibitory activity, HHL was used as the substrate and via monitoring the released hippuric acid by UV spectrophotometer, as described by Zheng, Li, Zhang, Ruan, and Zhang (2017). Briefly, 50 μl of samples extracts and 150 μl of 8.3 mM HHL solution were mixed and incubated at 37°C for 5 min. After that, 50 μl of 25 mU/ml ACE solution dissolved in the buffer of 100 mM sodium borate (containing 0.05 M sodium chloride and adjusted to pH 8.3) was added, incubated at 37°C for 60 min, and then 250 μl of 1 M HCl was added to terminate the reaction. A blank sample was prepared by replacing the inhibitor solution with 50 mM sodium borate buffer, and 1.4 ml of ethyl acetate was added to extract the hippuric acid by oscillating with a vortex mixer. Subsequently, the ethyl acetate mixture was centrifuged at 4,000 *g* for 5 min, finally, 1.0 ml of the top layer (containing hippuric acid extracted into ethyl acetate) was taken, and ethyl acetate was dried off by blast drier, 2 ml of deionized water was used to redissolved the hippuric acid, and the absorbance was read at 228 nm. The inhibitory rates (%) were calculated according to the following formula:

$$1-\left(A_{228\mathrm{sample}}-A_{228\mathrm{sample}\;\mathrm{blank}}\right)/\left(A_{228\mathrm{control}}-A_{228\mathrm{control}\;\mathrm{blank}}\right)]\times 100\%$$

### 2.12 Statistical analysis

All data were carried out in triplicate measurement, and the results were presented as mean value ± standard deviation. Results from each program were pooled, and statistical analysis was conducted using GraphPad Prism 6.0. The IC_50_ values for antioxidant activity and enzyme inhibition were calculated through fitting of the curve according to their best fit shapes based on at least four reaction points using Microsoft Excel.

## 3 RESULTS AND DISCUSSION

### 3.1 Total phenolic content and total flavonoid content

Phenolic and flavonoid, the most important plant secondary metabolites, have the potential health benefits to human body and display high correlations in the inhibition or management of many chronic diseases (Rahman, Costa de Camargo, & Shahidi, 2018). Previous studies indicated that the antioxidant action of *Allium* plants was strongly and positively correlated with the existence of polyphenols. Additionally, the well antioxidant activity showed by *Allium* depends on the chemical structure, the mass fraction of each single phenolic compound and the combinations and interactions among phenolic (Beretta et al., 2017). It was indicated that rosmarinic acid, p‐hydroxybenzoic acid, and protocatechuic acid all were identified as major phenolic compounds of *Allium* extract, and these compounds were contributors to the antioxidant activities (Mollica, Zengin, Locatelli, Picot‐Allain, & Mahomoodally, 2018). Content of phenolic compounds and flavonoids in different samples (Table 1) differed significantly (*p *< 0.05). It was proposed that the different polarity of solvent exert important effect in the recovery of polyphenols from different sources (Teruel, Garrido, Espinosa, & Linares, 2015). The data obtained in our study showed that the total phenolic and flavonoid content in *A. mongolicum* extracted by pure water was higher than that of methanol extract. Romero‐Diez et al. (2018) also reported that aqueous extract of sample plant showed better extracting power for phenolic compounds compared with methanol extract.

**Table 1:** Total phenolic and flavonoid content of *Allium mongolicum* extract in different solvent†

	Total phenolic content‡	Total flavonoid content‡
Aqueous extract	10.20 ± 0.09^a^	4.02 ± 0.02^a^
Methanol extract	7.50 ± 0.11^b^	3.13 ± 0.03^b^

*Notes*

Means followed by different letters (a, b) within the same column are significantly different (*p *< 0.05).

^†^Values are expressed as $\overset{¯}{X}$ ± *SD* of three replicates. ^‡^Total phenolic content was expressed as milligram gallic acid equivalents per gram dry weight, and total flavonoid content was expressed as milligram quercetin equivalents per gram dry weight.

### 3.2 Antioxidant activity

The evaluation on antioxidant activity of sample, a parameter to recognize nutritional health food from natural plants, provides valuable information with reference to health benefits and functional quality of raw material (Sharma et al., 2014). Our study used three antioxidant system (DPPH, ABTS, and FRAP) to assess the antioxidant potential of *A. mongolicum* extract as the result of one simple test assay for antioxidant was inappropriate and incomplete (Wang et al., 2016). The antioxidant capacity of the extracts got by different assays is shown in Table 2.

**Table 2:** The antioxidant properties of *Allium mongolicum* extract in different solvent†

	DPPH		ABTS		FRAP value (μmol Fe^2+^/g DW)
	IC_50_ ‡ (mg/ml)	TEAC§ (mg TE/g DW)	IC_50_ ‡ (mg/ml)	TEAC§ (mg TE/g DW)	
Aqueous extract	0.86 ± 0.01^b^	51.83 ± 0.77^a^	4.94 ± 0.03^b^	9.55 ± 0.17^a^	107.26 ± 5.48^a^
Methanol extract	0.98 ± 0.03^a^	49.53 ± 0.47^b^	6.08 ± 0.01^a^	7.74 ± 0.03^b^	85.29 ± 0.42^b^

*Notes*

Means followed by different letters (a, b) within the same column are significantly different (*p *< 0.05).

^†^All values were expressed as $\overset{¯}{X}$ ± *SD* of three replicates. ^‡^The IC_50_ values are defined as the extract concentration to inhibit 50% of free radicals under assayed conditions. ^§^The TEAC value is defined as milligram trolox equivalent of gram dry weight.

#### 3.2.1 DPPH and ABTS radical scavenging activity

DPPH and ABTS assays were used to evaluate antioxidant potential based on the ability to scavenge nonbiological stable free radicals (DPPH˙ and ABTS^+^˙). In the reaction process of DPPH assay, the absorbance of the reaction system decreases when antioxidant molecules encounter DPPH radicals. Thus, the DPPH scavenging capacities could be calculated by monitoring the change of absorbance in 517 nm. As shown in Table 2, the aqueous extract of *A. mongolicum* was able to interact with DPPH efficiently and quickly as the IC_50_ value was lower than methanol extract. Furthermore, a concentration‐dependent manner was found in the DPPH scavenging assay of these two extracts. It was noteworthy that the results of DPPH were comparable to *Allium elburzense* bulb extracts from different solvents (Safaeian, Zolfaghari, Aghaye‐Ghazvini, & Behnampour, 2017). Similar to DPPH scavenging, the same trend was found in ABTS assay: the aqueous extract showed better scavenging ability, and the percentage scavenging capacity of ABTS radical was dose‐dependent for both kind of extract. The Trolox equivalent antioxidant capacity of *A. mongolicum* extract in different solvents on DPPH scavenging tested in our study was better than the highest antioxidant part of *Allium scorodoprasum* L., while the TEAC on ABTS radical scavenging activity of AM was worse than that in *A. scorodoprasum* L. It is likely that the functional ingredients of AM and *A. scorodoprasum* L. to these different radicals were not quite the same (Mollica et al., 2018). As the literature indicated that the ability on scavenging free radicals against ABTS or DPPH were correlated with the concentration, chemical structures, and polymerization degree of organ antioxidants (Wang et al., 2016).

#### 3.2.2 Ferric reducing antioxidant power

**Figure 1:**
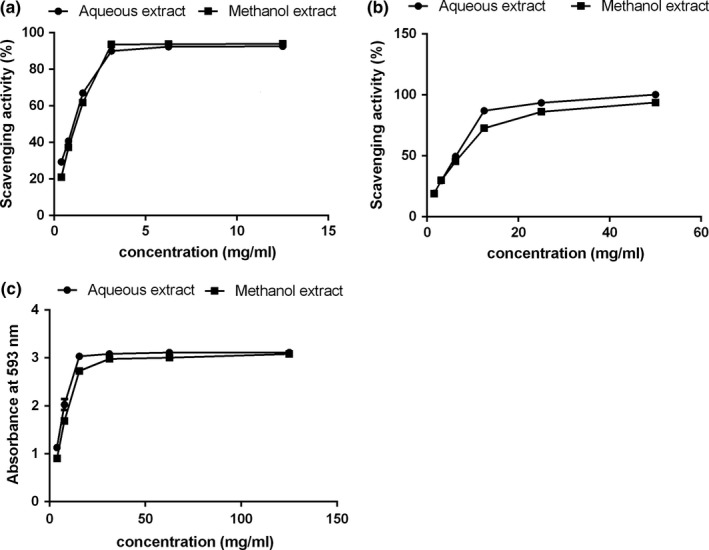
Antioxidant activity assessed by DPPH, ABTS, and FRAP assay

The FRAP assay evaluates the capacity of the antioxidants in a sample to reduce the ferric‐tripyridyltriazine complex (Fe^3+^‐TPTZ) to the colored ferrous‐tripyridyltriazine complex form (Fe^2+^‐TPTZ). The reducing power property of samples was mainly influenced by the electron transfer and hydrogen atom transfer mechanism (Oboh, Akinyemi, & Ademiluyi, 2013). The FRAP values of aqueous extract were higher than methanol extract by 107.26 μmol Fe^2+^/g DW, which indicates that the aqueous extract of *A. mongolicum* exhibited better reducing power activity. As we can see from Figure 1c, the absorbance increased as the concentration increased for each individual extract. The aqueous extract (125 mg/ml) presented ferric ion‐reducing activity, similar to that of methanol extract (3.15), with maximum absorbance of 3.22. The different content of phenolic compounds in samples could be used to explain the FRAP activity difference between these two kinds of extracts: the hydrogen donating ability of phenolic compounds might put the trigger on the transform of Fe^3+^ ferricyanide complex to the ferrous (Fe^2+^) form (Ndoye Foe et al., 2016).

### 3.3 α‐glucosidase inhibitory activity

α‐glucosidase plays a vital role in the breakdown of carbohydrate and polysaccharides into monosaccharides, which is of paramount importance in postprandial hyperglycemia in diabetes (Somtimuang, Olatunji, & Ovatlarnporn, 2018). Therefore, the inhibition on α‐glucosidase activity could be effective in the management of glucose releasing rate after food consumption.

**Figure 2:**
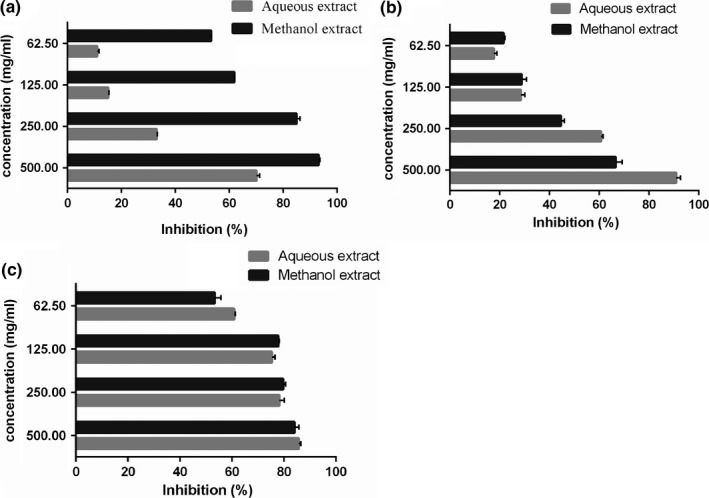
Inhibitory effects of *Allium mongolicum* extracts from methanol and aqueous on the activities of α‐glucosidase (a), pancreatic lipase (b), and Angiotensin I‐converting enzyme (c)

From the IC_50_ values (Table 3), the methanol extract exhibited the greater activity based on its potent inhibitory effect against α‐glucosidase, with IC_50_ values of 56.91 mg/ml, compared to aqueous extract. The percentage inhibitions of methanol extracts against α‐glucosidase were significantly higher than those of aqueous extract at all concentrations (Figure 2a). And the concentration of sample was responsible to the percent enzyme inhibition. It was proposed that the phenolic compounds could be used as α‐glucosidase inhibitor. Nevertheless, the aqueous extract of *A. mongolicum* had weaker inhibitory capacity against α‐glucosidase even though with high value of TPC and TFC than methanol extract. As the inhibitory efficiency of various individual phenolic components differ with others on account of the difference site of action, mechanism and binding affinities (Lalegani, Ahmadi Gavlighi, Azizi, & Amini Sarteshnizi, 2018). Hence, there may be some different phenolic compounds between these two kinds of extract except for flavonoid that display important roles in the activity of inhibition.

**Table 3:** IC_50_ values of *Allium mongolicum* extract in different solvent against α‐glucosidase, pancreatic lipase, and ACE†

	IC_50_ (mg/ml)† ^,^ ‡		
	α‐Glucosidase	Pancreatic lipase	ACE
Aqueous extract	330.82 ± 7.60^a^	179.48 ± 2.58^b^	18.57 ± 0.08^b^
Methanol extract	56.91 ± 0.38^b^	275.57 ± 13.60^a^	30.59 ± 3.70^a^

*Notes*

Means followed by different letters (a, b) within the same column are significantly different (*p *< 0.05).

^†^The IC_50_ values are defined as the extract concentration to inhibit 50% of enzyme activity. ^‡^Values are expressed as $\overset{¯}{X}$ ± *SD* of three replicates.

### 3.4 Lipase inhibitory activity

Pancreatic lipase, one of the most important digest enzyme, was responsible for digestion of dietary fat, what's more, these digestion products are stored in the adipose tissue causing diseases related to obesity (Ramirez et al., 2016). Thus, inhibition on the activity of pancreatic lipase is a valuable method for the treatment of overweight and obesity (Ercan & El, 2016). The lipase inhibitory effects of samples were measured by monitoring the hydrolysis of *p*‐nitrophenyl laurate (*p*NP laurate), which releases the yellow chromogen *p*‐nitrophenol (*p*NP). For comparison of inhibitory activity, the aqueous extract of *A. mongolicum* showed a good inhibitory activity, with IC_50_ values of 179 mg/ml. It can be seen from Figure 2b these two extracts both present lipase inhibitory activities in a concentration‐dependent manner, indicating a possible likeness in their action mechanism. There are studies indicated that the lipase inhibitory constituents could be better extracted from plants when water be the solvent, compared to methanol (Maqsood, Ahmed, Atique, & Malik, 2017). A great deal of edible *Allium* from *Liliaceae* family had been shown to exhibit favorable effects on antiobesity and oxidative stress, such as *Allium sativum* L. (Kim, Kim, Kim, & Om, 2013), *Allium fistulosum* L. (Sung, Yoon, Kim, Yang, & Kim, 2011), and *Allium cepa* L. (Kumari & Augusti, 2007).

### 3.5 ACE inhibitory activity

In this study, the IC_50_ values are summarized in Table 3. As the data showed, it was clear that both aqueous and methanol extract of *A. mongolicum* have significant potent inhibitory effect on ACE, and aqueous extract of *A. mongolicum* had stronger ACE inhibitory activity than methanol extract. In addition, both extracts tested showed a dose‐responsive inhibitory effect on ACE (Figure 2c). Flavonoids, the largest group of polyphenolic compounds, are viewed as an outstanding source of functional antihypertensive products. Studies about the relationship of structure–activity between inhibition of ACE activity and flavonoids proposed that the combination of substructures on the flavonoid skeleton, including the catechol group in the B‐ring, the double bond between C2 and C3 at the C‐ring, and the cetone group in C4 at the C‐ring, increases the ACE inhibitory activity of the flavonoids (Irondi, Agboola, Oboh, & Boligon, 2016). Furthermore, it was indicated that free hydroxyl groups of phenolic compounds were important structural moieties to chelate the zinc ions, which could inactive the activity of ACE (Chen, Lin, Lin, & Hsu, 1992). ACE inhibitors have been widely developed to prevent angiotensin II production in renal dysfunction, and it was indicated by literatures that garlic had outstanding blood pressure‐lowering properties without significant side effects (Ramesar, Baijnath, Govender, & Mackraj, 2008). As the scale of hypertensive population is on the rise in developing countries, it is necessary to track further studies in this area.

## 4 CONCLUSION

**Figure 3:**
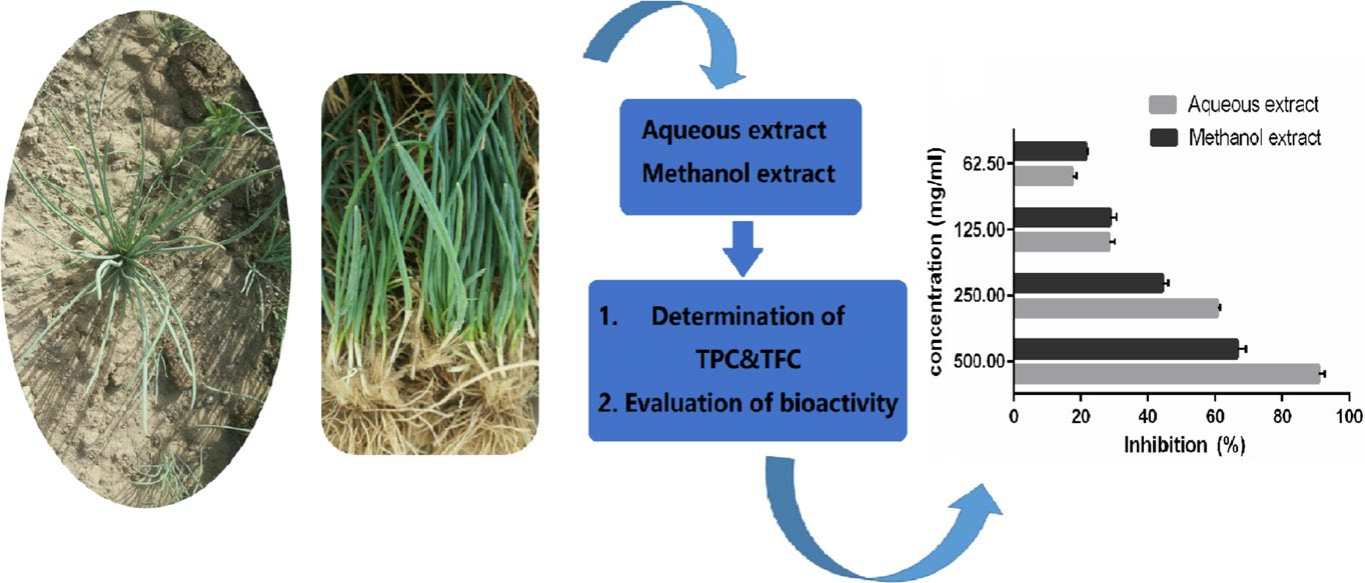
The outline of this study

The present study evaluated the content of total phenolic and total flavonoid and investigated the potential antioxidant of *A. mongolicum* extracted from different solvents (water and methanol), focusing on the inhibitory effects of α‐glucosidase, pancreatic lipase, and ACE. The graph outline is shown in Figure 3. The results suggested that water may be an excellent extraction solvent for *A. mongolicum* to extract chemical components. In addition, the aqueous extract of *A. mongolicum* could be used as an antioxidant, which contains highly contents of phenolic and flavonoid compound, and had better potential to inhibit ACE as well as pancreatic lipase. In summary, this study offered essential scientific support to the application of *A. mongolicum* as nutraceutical for dietary management of diabetes, obesity, and hypertension. Further studies are required to characterize active constituent(s) responsible for the observed activities and to elucidate the detailed mechanism of actions at the level of cellular and molecular.

## CONFLICT OF INTEREST

The authors declare that there are no conflict of interests in the publication of the manuscript.

## AUTHOR CONTRIBUTIONS

The authors’ responsibilities were as follows—Wang, W.Y. and Zhang, X.F. conceived and designed this study, Wang, W.Y., Li, J.and Wang, X.K. participated in the experiments and analyzed the data, Wang, W.Y., Zhang, H.Z, Fan, J.M. and Zhang, X.F. interpreted the data and wrote the manuscript. All authors discussed the contents of the manuscript and approved the submission.

## ETHICAL STATEMENT

This study does not involve any human or animal testing.
